# Rectal cancer management: the essential role of magnetic resonance imaging in neoadjuvant therapy

**DOI:** 10.1590/0100-3984.2024.57.e6

**Published:** 2024-09-23

**Authors:** Aley Talans, Giuseppe D’Ippolito

**Affiliations:** 1 Instituto de Radiologia do Hospital das Clínicas da Universidade de São Paulo (InRad/HC-FMUSP), São Paulo, SP, Brazil. Email: aleytalans@gmail.com; 2 Department of Diagnostic Imaging, Escola Paulista de Medicina da Universidade Federal de São Paulo (EPM-Unifesp), São Paulo, SP, Brazil. Email: giuseppe_dr@uol.com.br

The evaluation of rectal tumors by magnetic resonance imaging (MRI) has made one of the greatest contributions to the management of the disease over the last two decades. Its importance has grown because of the high prevalence of colorectal cancer, which is the third most common cancer and the second leading cause of cancer-related death^(1)^, as well as because of improvements in the technique and in the quality of the images obtained, especially in the context of rectal adenocarcinoma, which accounts for approximately one-third of these tumors, with a worrisome increase in incidence among younger patients^(2)^.

In recent years, neoadjuvant treatment became a crucial strategy in the treatment of rectal cancer, especially in cases of locally advanced disease^(3)^. In that context, MRI plays a role not only in evaluating patients with advanced carcinoma but also in selecting patients with early-stage tumors, which have a better prognosis and specific treatments, compared with locally advanced tumors. In the latter, MRI is also crucial in assessing the response to neoadjuvant treatment. The American Society of Abdominal Radiology^(4)^ adopts the following response categories, as were also used in the Organ Preservation for Rectal Adenocarcinoma trial^(5)^: complete response; near-complete response; and incomplete response. In the first two categories, nonsurgical treatment (watchful waiting) can be considered, in order to preserve the organ and improve quality of life. This strategy was initially proposed by Habr-Gama et al.^(6)^ and represented a paradigm shift in the treatment of rectal cancer. If surgical treatment is the chosen option, MRI works as a roadmap for the surgery to be performed.

A review article recently published in Radiologia Brasileira, entitled “Restaging magnetic resonance imaging of the rectum after neoadjuvant therapy: a practical guide”^(7)^, emphasized the technical aspects, interpretation, and description of post-neoadjuvant treatment MRI. The authors discuss some protocol aspects, such as the suggestion of bowel preparation with a micro-enema to reduce rectal air susceptibility artifacts in diffusion-weighted sequences, and corroborate the lack of benefit in the use of rectal contrast or an endorectal coil^(8)^, as demonstrated in the literature, as well as the lack of superiority of the use of intravenous contrast^(7)^. They also emphasize the importance of a baseline MRI study, which is important not only for comparison purposes but also for correct evaluation of the tumor site and the identification of any mucinous component before treatment (indicating a worse prognosis, as opposed to the appearance of mucin after treatment, which indicates a response). In the post-treatment evaluation, some institutions adopt the tumor regression grade (TRG) classification, grading the proportion of fibrosis/viable tumor. It is worth noting that there is a good correlation between the post-neoadjuvant TRG classification determined by MRI correlates well with the prognosis, as does, to a limited extent, the TRG classification determined by pathology. However, it is important to highlight that there is a significant interobserver variability in the definition of the MRI-based TRG determined by a radiologist^(9)^.

In the context of post-neoadjuvant therapy, the evaluation of mesorectal lymph nodes is no longer a cornerstone in the management of these patients-total excision of the mesorectum, performed properly, removes those lymph nodes^(10)^. In contrast, lateral pelvic lymph nodes should be adequately described, given that they are not included in the standard resection. Radiologists can instead focus their attention on accurate assessments of extramural vascular invasion and the mesorectal fascia, both of which are more accurately evaluated with MRI and have important prognostic value and affect the risk of local disease recurrence^(11)^. The timing of the MRI reassessment, classically performed 8-12 weeks after the initiation of neoadjuvant therapy, has also been discussed, with some surgeons recommending an early interval reassessment, given that most of the response occurs at the beginning of treatment^(12)^.

The treatment of rectal tumors has been constantly updated: initially with the adoption of neoadjuvant therapy, including radiosensitizing chemotherapy and radiotherapy, as well as nonsurgical treatment for selected cases, and more recently with total neoadjuvant therapy, which adds systemic chemotherapy to “classical” neoadjuvant therapy, both before surgery^(8,13)^. This latter scheme has shown lower rates of local recurrence, longer disease-free survival, improved compliance with therapy, and better complete response rates^(14)^.

In summary, in their article, Horvat et al.^(7)^ develop a well-structured and didactic guide for the MRI examination and its report, based on a rational and updated review of the literature, to assist radiologists in their essential participation in the approach to the patient with rectal carcinoma. In addition, the figures provided in the article, which illustrate how to interpret MRI examinations, are welcome and useful, especially to familiarize the general radiologist with the main imaging aspects of this type of lesion, which presents particularities due to having been submitted to neoadjuvant therapy.

The future holds many challenges, including the widespread adoption of specific and abbreviated MRI protocols and new rectal cancer treatment guidelines, which are still restricted to only the most up-to-date facilities. Individualized indication of neoadjuvant treatment, making it possible to select the patients who will better respond to a given therapy (upfront surgery, neoadjuvant therapy with or without organ preservation, or immunotherapy), is of crucial importance for advances in rectal cancer treatment.
